# The Lymphatic System in Mammary Gland Biology and Breast Cancer

**DOI:** 10.1007/s10911-024-09558-0

**Published:** 2024-03-17

**Authors:** Traci R. Lyons, Esta Sterneck

**Affiliations:** 1 Division of Medical Oncology, Department of Medicine, University of Colorado Anschutz Medical Campus, Aurora, CO USA; 2 Young Women’s Breast Cancer Translational Program, University of Colorado Cancer Center, Aurora, CO USA; 3 Laboratory of Cell and Developmental Signaling, Center for Cancer Research, National Cancer Institute, Frederick, MD USA

The proper functioning of an organism and any organ within it requires the concerted and harmonic cooperation of all its diverse components. One of these essential parts is the lymphatic vasculature, which has for the longest time lingered in the shadow of its more visible counterpart – the blood vasculature. However, recent substantial developments and improvements in molecular approaches and imaging tools are beginning to shed light onto the details of anatomy and functions of the lymphatic system in health and disease, especially cancer [1]. Tumor development and progression are intricately connected with the functions of the lymphatics as is their contribution to patient treatment and recovery [2].

In this special issue of The Journal of Mammary Gland Biology and Neoplasia we have collected contributions on the involvement of the lymphatic system in normal mammary gland physiology and breast cancer metastasis as well as cancer patient recovery. There are four articles which span the range from clinical implications of axillary lymph node dissection (Kinney et al. [3]) to the impact of lifestyle factors such as diet and duration of lactation (Balema et al. [4]), to basic science implicating lymphatic secretions in attraction of breast cancer cells (Giotopoulou et al. [5]) and finally a comprehensive literature review of lymphatic remodeling that occurs during mammary development and breast cancer progression (Dahms and Lyons [6]).

As described in the article by Dahms and Lyons, the lymphatic vasculature is remodeled during each stage of mammary gland development including puberty, pregnancy, and involution. These processes have been characterized through both imaging as well as histological analyses. Additionally, the contribution of immune-suppressive macrophages is reviewed and several signaling axes that are in common between normal development and cancer progression overall suggesting that the similarities between mammary lymphatics and tumor associated lymphatics could be exploited to further understand and/or prevent breast cancer metastasis.

In support of this, the manuscript by Fuxe and colleagues identifies mechanisms by which TGFβ stimulates the migration of mesenchymal breast tumor cells to the lymphatics, which may contribute to lymphatic metastasis. TGFβ is a well-known mediator of mammary gland development. The study by Giotopoulou et al. identifies chemokines that are associated with poor prognosis in patients as mediators of tumor cell migration which may facilitate interaction and intravasation into lymphatics to promote tumor cell dissemination. Of particular interest is the RNASeq analysis of the lymphatic endothelial cells after exposure to TGFβ; this unique data set, when made publicly available, will provide a valuable resource to the research community and may reveal additional pathways important for lymphatic remodeling and metastatic spread through the lymphatics.

In the article presented by Woodward and colleagues, the contributions of diet and the primary function of the mammary gland, lactation, are investigated for potential roles in inflammatory breast cancer (IBC). This highly aggressive subtype of breast cancer involves invasion of skin lymphatics by tumor cell emboli and links to obesity and breastfeeding have previously been proposed as contributing to the unique biology of IBC. The study makes use of in vivo imaging of lymphatic pulsing in mice to investigate how high fat diet (HFD) and duration of lactation modulate lymphatic changes in a model of IBC. Their results suggest that HFD drives mammary lymphatic function in a manner that is independent of duration of lactation as well as parity. Furthermore, they identify a correlation of monocyte-derived cells in the tumor microenvironment with HFD. Since a normal role for lymphatics is the transport of lipids, these findings may also suggest that a systemic excess of fats may modulate lymphatic function in the mammary gland.

Finally, the manuscript by Singhal and colleagues utilizes lymphatic imaging in the form of preoperative lymphography to determine linear versus non-linear patterns of lymphatics in the upper extremities of breast cancer patients. Their results identify not only risk factors for development of non-linear patterns, but also suggest that non-linear patterns are associated with a higher risk for lymphedema after axillary lymph node dissection. For breast cancer survivors, lymphedema can be a lifelong condition that severely impedes quality of life, highlighting the importance of the outcome of these findings which is prophylactic prescription of compression sleeves for patients with non-linear patterns of lymphatics. This clinically relevant discovery further highlights the importance of understanding tissue draining lymphatic remodeling for both developmental biology and organ functions as well as for tumor biology and patient care.

In conclusion, this collection of articles highlights the wide variety of aspects by which the lymphatics play important roles in normal physiology and cancer and creates new foundations for additional investigations and discoveries to further improve our understanding and management of lymphatics in pathologies.
